# Congenital Cytomegalovirus Infection Presenting with Hyperbilirubinemia and Splenomegaly in a Term Infant with Trisomy 21

**DOI:** 10.1155/2020/2534629

**Published:** 2020-02-12

**Authors:** Kate Wilson, Lindsay Ellsworth, Megan H. Pesch

**Affiliations:** ^1^Division of Neonatal Perinatal Medicine, Department of Pediatrics, University of Michigan Medical School, Ann Arbor, MI, USA; ^2^Division of Developmental and Behavioral Pediatrics, University of Michigan Medical School, Ann Arbor, MI, USA; ^3^Center for Human Growth and Development, University of Michigan, Ann Arbor, MI, USA

## Abstract

Congenital cytomegalovirus infection (cCMV) is very common, yet the presentation can be varied, making the diagnosis challenging. However, early diagnosis for treatment with medication in symptomatic cases within the first month of life is critical. Hyperbilirubinemia and splenomegaly are less common manifestations at birth and may be overlooked in the setting of other symptoms, especially in a critically ill neonate. We present a case of a term infant with trisomy 21 who presented with isolated hyperbilirubinemia and splenomegaly and was later diagnosed with congenital CMV.

## 1. Introduction

Congenital cytomegalovirus (cCMV) infection is the most common congenital infection worldwide [1], which disproportionately affects infants in developing countries [2] and those of lower socioeconomic status [3]. This infection affects 1 in every 150–200 live births in developed countries and 1 in 20–100 live births in developing countries [2, 4, 5]. Infants with congenital CMV can experience hearing loss, vision loss, intellectual disability, cerebral palsy, epilepsy, autism, and developmental delays [6–8]. Yet there is low public and healthcare provider awareness about this preventable disease [9–13]. Congenital CMV is best identified early, ideally in the first month of life [14], which opens the door for treatment with medication which has been shown to impact developmental outcomes and preserve hearing in symptomatic cases [15]. However, recent work has shown that cCMV is underdiagnosed [16]. This may be because infants born with cCMV can present with a variety of subtle systems, with only the minority presenting as the classic “blueberry muffin baby” [17]. Furthermore, many early signs of congenital CMV are common in the newborn period in even healthy infants (e.g., jaundice and petechiae) [18]. A high degree of clinical suspicion on the part of the pediatrician is necessary to diagnose congenital CMV. The diagnosis of congenital CMV may be even more likely to be overlooked in medically complex infants, or those with genetic disorders. We present a case of an infant with trisomy who presented with hyperbilirubinemia and splenomegaly. The clinical course of this infant was likely prolonged due to his presentation in the setting of a known genetic disorder.

## 2. Case Presentation

A term African American male infant was born at 37 weeks 5/7 days to a 40-year-old G_4_P_3_ mother by repeat caesarian section after spontaneous onset of labor. The pregnancy was complicated by late prenatal care in the third trimester. Prenatal genetic screening returned concerning for trisomy 21. At birth, Apgar scores at 1 and 5 minutes were 9 and 9, respectively. Per the growth chart for boys with Down syndrome, he was microcephalic at the 7th percentile, otherwise well grown. Physical exam was notable for Down syndrome facial features, systolic murmur, splenomegaly, jaundice, and scattered petechiae.

Laboratory evaluation at his birth hospital confirmed the clinical suspicion for Down syndrome with karyotype 47 XY, +21. Echocardiogram showed a moderate atrial septal defect. Thyroid-stimulating hormone elevated to 11.31 *μ*IU/mL with a free T4 elevated to 2.68 ng/dL concerning for congenital hypothyroidism. Workup for clinically apparent petechiae and jaundice revealed a platelet count of 45,000/*μ*L on day of life 1 and total bilirubin of 10.4 mg/dL with a direct component level of 4.6 mg/dL on day of life 3. The infant's blood type was O+, and direct antigen testing was negative. Peripheral blood smear revealed anisopoikilocytosis, eosinophils, and circulating blasts. Due to ongoing splenomegaly on examination, abdominal ultrasound was obtained confirming splenomegaly measuring 6.4 × 1.9 × 5.6 cm. Toxoplasma, rubella, cytomegalovirus (CMV), and herpes simplex virus (TORCH) serologic evaluation was obtained with results pending at time of transfer. Due to persistent lab abnormalities of unclear etiology, the infant was electively transferred to a quaternary-level neonatal intensive care unit for additional subspecialty evaluation.

Upon transfer, Pediatric Gastroenterology, Endocrinology, and Hematology/Oncology were consulted as laboratory studies revealed a persistent direct hyperbilirubinemia, thrombocytopenia, and hypothyroidism. Further evaluation of direct hyperbilirubinemia, thrombocytopenia, and hepatosplenomegaly was pursued while initiating levothyroxine for treatment of congenital hypothyroidism. Repeat abdominal imaging visualized the gallbladder and common bile duct and normal blood flow within the vasculature. He was noted to have transaminitis with ALT and AST peaking at 350 IU/L and 214 IU/L, with normal synthetic liver function, GGT, and alpha-1 antitrypsin levels. Bacterial infectious workup for sepsis with blood and urine cultures was negative. Initial TORCH evaluation completed at the birth hospital returned negative for toxoplasmosis, rubella, and herpes simplex virus, but with an inconclusive CMV IgG of 2.27 U/mL (detected) and undetected CMV IgM. Metabolic evaluation for cholestasis including urine organic acids, plasma amino acids, pyruvate, and lactate returned negative. Evaluation of a malignant hematologic process given history of blasts was negative based on serial blood counts and review of peripheral smears. By day of life 21, his thrombocytopenia, direct hyperbilirubinemia, and transaminitis persisted, prompting further viral studies including Epstein–Barr virus, cytomegalovirus, adenovirus, herpes simplex virus, and hepatitis C to be ordered. Serum cytomegalovirus (CMV) DNA polymerase chain reaction (PCR) returned positive at 1697 IU/mL (detectable limit of quantification > 50 IU/mL), confirming the diagnosis of congenital CMV.

Pediatric Infectious Diseases was consulted given positive CMV PCR and concern for congenital cytomegalovirus. Review of TORCH serologies noted a negative CMV IgM and a minimally elevated CMV IgG at 2.7. He was strictly formula fed and never received a blood transfusion throughout his hospital course, thus making postnatally acquired CMV infection unlikely. Further evaluation was obtained to determine the extent of his cCMV infection. Cranial ultrasound revealed lenticulostriate mineralizing vasculopathy, consistent with a TORCH infection. Ophthalmology exam showed no evidence of chorioretinitis. He passed his newborn hearing screen bilaterally. Given the infant's constellation of clinical findings with his laboratory abnormalities, he was determined to be symptomatic for cCMV infection and started on a 6-month course of oral valganciclovir 16 mg/kg twice a day. Labs including complete blood count and comprehensive metabolic panel were obtained weekly to monitor for medication side effects. Neutropenia developed and remained stable with an absolute neutrophil count ranging 0.5–0.8 K/*μ*L. Thrombocytopenia persisted but remained >50,000/*μ*L without transfusions. His direct hyperbilirubinemia remained stable at 4.8 mg/dL, and his transaminitis improved. On day of life 45, the infant was discharged to home with his mother and instructed to follow-up with several pediatric subspecialties including Infectious Disease and Audiology. Written consent was obtained from the patient's mother to share this information in the form of a case report.

## 3. Discussion

Congenital cytomegalovirus infection is the most common congenital viral infection, affecting one in every 150 to 200 pregnancies in the United States [16]. However within the United States, African American infants have the highest cCMV prevalence, 9.5 per 1000 live births, compared to other racial and ethnic groups [3]. The majority of infants with cCMV do not display symptoms at birth. Approximately 10–15% of infants will present with symptoms of cCMV at birth. Currently, there is no standard definition for symptomatic disease other than the presence of multiple symptoms. Presenting symptoms are presented in Table 1 and include direct hyperbilirubinemia, thrombocytopenia, and splenomegaly [14, 19]. Early identification of infants with cCMV is important as the optimal treatment window is prior to one month of age [15]. However, the wide clinical spectrum of cCMV presentations creates a diagnostic challenge. Without universal screening programs, which are not commonplace in the United States [21], cCMV is often diagnosed late, as in this case, or missed altogether [22]. Relying on physician suspicion alone may lead to this diagnostic and treatment delay as symptoms of cCMV may appear to be explained by other co-occurring diagnoses. In this case, the patient's direct hyperbilirubinemia and thrombocytopenia were attributed to his other diagnoses, primarily his trisomy 21 and associated congenital hypothyroidism. Healthcare systems may improve their cCMV screening and diagnostic rates by increasing clinician awareness through in-service lectures and presentations, as well as standardizing practice guidelines for screening, testing, and treatment of cCMV. An example of such a guideline is available from authors upon request.

Furthermore, cCMV is a condition for which there are treatments including both pharmacologic and developmental supports regardless of whether an infant is deemed symptomatic or asymptomatic at birth [23]. Early identification of cCMV is extremely important to implement these supports to optimize a child's developmental outcomes. Universal screening cCMV would allow for increased detection, even for infants who are born asymptomatic, who are, in fact, still at increased risk of later hearing loss and neurodevelopmental sequelae [24]. Treatments available include a six-month course of oral valganciclovir which has been shown to improve both hearing and developmental outcomes in symptomatic infants [15]. Of note, there are ongoing trials in the United States investigating pharmacologic treatment in asymptomatic children that appear promising. In addition, frequent monitoring of hearing and vision and heightened developmental surveillance can identify any deficits or delays that may arise, allowing for more prompt intervention [19–21].

## 4. Conclusion

Congenital CMV is a common congenital infection that is underrecognized. The clinical spectrum of cCMV infection varies widely presenting challenges for a timely diagnosis. Infants with congenital diseases or genetic disorders such as trisomy 21 can have co-occurring infections, and thus, it is important to consider common associated etiologies first. However, testing for cCMV should be done in cases of unexplained hearing loss, thrombocytopenia or petechiae, direct hyperbilirubinemia, microcephaly, and growth restriction [20, 25]. A clinical practice guideline and standardization of the screening and diagnostic testing for cCMV could result in increased timely diagnoses and treatment for all infants.

## Figures and Tables

**Table 1:** Common signs and findings suggestive of a congenital cytomegalovirus infection [14, 19, 20].

Physical exam findings	Laboratory test findings	Imaging/additional assessments
(1) Intrauterine growth restriction	(1) Thrombocytopenia	(1) Head ultrasound imaging
(2) Small for gestational age	(2) Direct hyperbilirubinemia	(i) Ventriculomegaly
(3) Microcephaly	(3) Elevated transaminases	(ii) Periventricular echogenicity
(4) Seizures	(4) Anemia with hemolysis	(iii) Intracerebral calcifications
(5) Hypotonia	(5) CSF pleocytosis, elevated protein	(iv) Cortical malformations
(6) Poor sucking/feeding		(v) Cerebellar malformations
(7) Unexplained hydrops		(2) Ophthalmology exam
(8) Hepatomegaly		(i) Chorioretinitis
(9) Splenomegaly		(3) Hearing loss on audiology exam
(10) Petechiae		(i) Sensorineural hearing loss
(11) Jaundice		(4) Fetal ultrasound with echogenic bowel

## Abbreviations

CMV: Cytomegalovirus
TORCH: Toxoplasma, rubella, cytomegalovirus, and herpes simplex virus.
